# The Effects of the COVID-19 Pandemic on the Follow-Up of Patients with Age-Related Macular Degeneration in a Tertiary Hospital in London, the UK

**DOI:** 10.3390/jcm14186497

**Published:** 2025-09-15

**Authors:** Inés López-Cuenca, Lorenzo Fabozzi, Saad Younis, Ahmad Ali, José M. Ramírez, Maria Francesca Cordeiro, Rosa de Hoz

**Affiliations:** 1 Ramon Castroviejo Institute for Ophthalmic Research, Complutense University of Madrid (ROR 02p0gd045), 28040 Madrid, Spain; inelopez@ucm.es (I.L.-C.); ramirezs@med.ucm.es (J.M.R.); 2 Health Research Institute of the Hospital Clínico San Carlos (IdISSC) (ROR 014v12a39), 28040 Madrid, Spain; 3 Department of Immunology, Ophthalmology and ENT, Faculty of Optics and Optometry, Complutense University of Madrid, 28040 Madrid, Spain; 4 Western Eye Hospital, Imperial College Healthcare NHS Trust, London NW1 5QH, UK; lorenzo.fabozzi@nhs.net (L.F.); saad.younis@nhs.net (S.Y.); ahmad.ali7@nhs.net (A.A.); 5 University Hospitals Sussex NHS Foundation Trust, Worthing BN11 2DH, UK; 6 Department of Immunology, Ophthalmology and ENT, School of Medicine, Complutense University of Madrid, 28040 Madrid, Spain; 7 Imperial College Ophthalmic Research Group (ICORG) Unit, Imperial College London, London NW1 5QH, UK

**Keywords:** age-related macular degeneration, AMD, COVID-19, follow-up delay, anti-VEGF

## Abstract

**Background/Objectives:** Adherence to medical appointments is crucial for managing age-related macular degeneration (AMD). The COVID-19 pandemic disrupted healthcare services, potentially affecting disease control. This study aimed to assess the impact of the pandemic on appointment adherence and disease progression in AMD patients at the Western Eye Hospital in London. **Methods:** Patients were divided into two groups: those who attended appointments on time and those who experienced delays (*n* = 100 per group). We compared disease progression using demographic data, best-corrected visual acuity (BCVA), and macular thickness measured by OCT, extracted from medical records. **Results:** In patients without delays, BCVA remained stable pre- and post-COVID-19, although significant changes in macular thickness were observed in the central (C0), superior (S1, S2), nasal (N1), and inferior (I1, I2) macular sectors. In contrast, patients with delayed appointments showed a significant increase in N1 macular thickness from 324.00 (304.00–358.00) pre-COVID-19 to 337.50 (305.50–375.50) post-COVID-19 (*p* = 0.030). Post-COVID-19, patients without delays had significantly better BCVA and thinner N1 macular thickness than those with delays. A positive correlation was found between the length of appointment delays and increased macular thickness in S1 and I2 sectors. **Conclusions:** Timely follow-up is essential in AMD management. Appointment delays during the COVID-19 pandemic were associated with increased macular thickness and worse visual outcomes, highlighting the importance of maintaining continuity of care even during healthcare disruptions.

## 1. Introduction

The COVID-19 pandemic was caused by the new betacoronavirus (SARS-CoV-2) and can lead to pneumonia, severe acute respiratory syndrome (SARS), and even death [1]. This has resulted in unprecedented disruption to healthcare services worldwide, particularly affecting medical specialties that require close patient contact, such as ophthalmology. The first case of COVID-19 in the United Kingdom (UK) was reported on the 31 January 2020, and in March 2020, the COVID-19 became a public health concern, forcing the UK government to declare the first severe lockdown from 23 March to August 2020 [2].

SARS-CoV-2 is highly transmissible, and ophthalmology is considered a high-risk specialty due to the necessity of face-to-face interactions and frequent contact with shared examination equipment, both of which increase the potential for viral transmission [3,4,5,6,7,8]. To mitigate the risk of infection among healthcare workers, protective measures and enhanced hygiene protocols were implemented [9,10,11]. Nevertheless, it was recommended that non-urgent ophthalmic procedures be postponed in order to minimise exposure [11]. In England, the impact of these restrictions was evident in general practice consultations, where the proportion of face-to-face visits declined from 80% in February 2020 to 47% in April 2020 [12]. Consequently, ophthalmic practices were thoroughly reorganised during the first wave of the pandemic to ensure the safety of both clinicians and patients [9]. Additionally, misinterpretations of government guidelines—stating that healthcare services were closed for non-COVID-19 care, including emergency treatments—further contributed to decreased hospital attendance [13].

Neovascular age-related macular degeneration (nAMD) is a leading cause of central vision loss in older adults and requires regular intravitreal anti-VEGF therapy to preserve visual function [14]. Despite the need for continuous monitoring and treatment, the COVID-19 pandemic disrupted ophthalmic care globally, leading to widespread delays in routine follow-up and treatment, particularly among vulnerable populations with comorbidities [14]. Given the increased mortality risk associated with COVID-19 in elderly patients and those with multiple comorbidities, AMD patients face heightened risks concerning both their overall health and functional visual prognosis [9,13].

The Western Eye Hospital (WEH), part of the Imperial College NHS Trust in London, serves as a tertiary referral centre for degenerative and vascular retinal diseases, including AMD. During the first wave of the pandemic, the number of consultations in medical retina clinic declined significantly, from 1345 patients seen between 23 March and 3 May 2019, to only 510 patients in the same period in 2020 [10].

To manage care during the pandemic, the medical retina clinic at WEH implemented a risk-based protocol, categorising patients by disease severity. NHS England notified patients of cancellations, and clinicians conducted telephone triage to assess conditions. Those without COVID-19 symptoms were encouraged to attend, while patients who declined were rescheduled within 4–8 weeks and advised to seek emergency care if their vision worsened [10].

Although previous studies have described the general impact of the pandemic on healthcare delivery, limited evidence exists regarding how delayed visits specifically affected clinical outcomes in patients with neovascular AMD undergoing anti-VEGF therapy. This gap is particularly significant given the progressive nature of the disease and the time-sensitive nature of its treatment [15].

This study investigates the consequences of postponed appointments during the first wave of the pandemic at the Western Eye Hospital (London), comparing disease progression between patients who maintained their scheduled visits and those who experienced delays. The findings aim to inform future strategies for managing nAMD during public health crises and highlight the importance of uninterrupted care in chronic retinal conditions.

## 2. Materials and Methods

Patients were selected from the Macula Clinic at the Western Eye Hospital (WEH) using Medisoft Ophthalmology (Medisoft Limited, Leeds, UK), an electronic medical record system. Two distinct patient groups were identified based on their adherence to scheduled medical appointments.

- No-Delay Group: This group comprised patients who strictly adhered to their scheduled appointments. For analysis, two specific time points were selected—the last appointment before the first London lockdown (23 March–30 August 2020) and the first appointment following this period.
- Delay Group: This group included patients who either missed, refused, or postponed their medical appointments due to fear of COVID-19 infection. For these patients, the last appointment before the first lockdown (23 March–30 August 2020) and the first appointment during the recovery phase were selected for analysis.

For both groups, best-corrected visual acuity (BCVA) assessments and macular optical coherence tomography (OCT) (Heidelberg Engineering, Heidelberg, Germany) imaging were conducted at the selected pre- and post-lockdown appointments.

### 2.1. Patient Inclusion

From the total database of 3201 patients, we applied a stratified sampling approach by manually reviewing clinical records and classifying patients into two groups: those with appointment delays and those without. From each group, we selected 100 patients to create two balanced subsamples, enabling a controlled comparative analysis. This strategy ensured equal group sizes, which facilitates statistical testing and improves the interpretability of results. The sample size was chosen to provide sufficient statistical power while maintaining feasibility for detailed data review.

### 2.2. Inclusion and Exclusion Criteria

Patients were eligible for inclusion if they met the following criteria:

1. Confirmed diagnosis of AMD with at least one visit before March 2020, during which a macular optical coherence tomography (OCT) scan was performed.
2. Having had at least one face-to-face appointment between June 2020 and September 2021.
3. Having had at least one macular OCT scan conducted during the “recovery phase” of the COVID-19 pandemic.
4. Missed or delayed at least one scheduled face-to-face appointment in the macula clinical unit due to concerns about COVID-19 infection during the first lockdown.

Patients were excluded if they met any of the following conditions:

1. No AMD diagnosis (e.g., patients followed up for other macular diseases such as branch retinal vein occlusion or diabetic macular oedema).
2. Follow-up conducted in another ophthalmology centre.
3. The presence of other ocular or systemic conditions that could negatively influence disease progression.

Patient inclusion was assessed by the research team. Demographic and clinical data were collected from medical and nursing records, including age, sex, race, or ethnicity; arterial hypertension; diabetes; hypercholesterolemia status; type of age-related macular degeneration (AMD); and the affected eye.

The pre-COVID-19 variables recorded included the type of AMD, history of previous cataract surgery, the interval (in weeks) between visits prior to the COVID-19 pandemic, best-corrected visual acuity (BCVA) measured on the logarithm of the minimum angle of resolution (logMAR) scale, and macular thickness across different sectors as assessed by optical coherence tomography (OCT) using the Heidelberg Spectralis (Heidelberg Engineering, Heidelberg, Germany) in accordance with the Early Treatment Diabetic Retinopathy Study (E.T.D.R.S.) protocol. All patients at WEH received intravitreal injections following a treat-and-extend (T&E) protocol. In addition, the number and type of prior intravitreal injections (IVIs), as well as the interval (in weeks) until the next scheduled appointment, were documented

**Figure 1:**
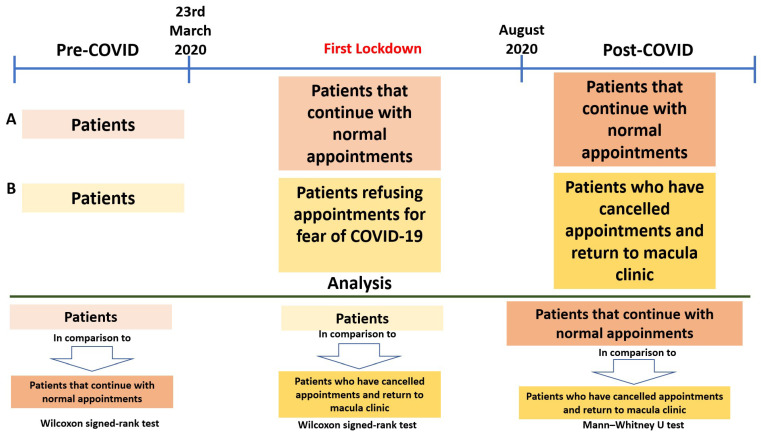
Study groups and analysis carried out. Patient attendance timeline before, during, and after the COVID-19 pandemic, highlighting the impact of lockdown on appointment adherence. Comparative analysis between patient groups was performed using the Wilcoxon signed-rank test and the Mann–Whitney U test.

Post-COVID-19 visit data included BCVA, macular thickness, the necessity for IVI and its type, history of cataract surgery, and the number of weeks until the subsequent visit. Figure 1 illustrates the different study groups and the corresponding analyses performed.

### 2.3. Statistical Analysis

Statistical analysis was conducted using SPSS 25.0 (SPSS Inc., Chicago, IL, USA). Within-group comparisons of pre- and post-COVID-19 variables were performed using the Wilcoxon signed-rank test, which is appropriate for dependent data that do not follow a normal distribution. Post-COVID-19 variable comparisons between groups were analysed with the Mann–Whitney U test. Categorical variables were assessed using the chi-squared test, and Spearman’s rank correlation analysis was applied to evaluate potential associations between appointment delays (in days) and changes in BCVA and retinal thickness measured by OCT, as the data did not follow a normal distribution. A *p*-value ≤ 0.05 was considered statistically significant.

## 3. Results

In this study, a total of 200 patients were selected. Of these, 100 patients attended their appointments promptly, while the remaining 100 experienced delays during the COVID-19 lockdown. These delays were attributed to either missed appointments or rescheduled visits due to concerns about the pandemic. A review of ocular medical records was conducted, and one eye was randomly selected from each patient, resulting in a total of 200 eyes diagnosed with age-related macular degeneration (AMD).

The study cohort comprised 79 male patients (39.5%) and 121 female patients (60.5%), with a median age of 80 years (range: 74.00–86.00). Regarding systemic comorbidities, arterial hypertension was present in 135 patients (67.5%), diabetes mellitus in 31 patients (15.5%), and hypercholesterolemia in 112 patients (56%). Ethnic distribution included 84 British patients (42%), 6 African patients (3%), 70 patients (35%) classified as belonging to another race or ethnicity, and 40 patients (20%) whose ethnicity was not stated.

All 200 patients (100%) were diagnosed with wet AMD. Analysis of ocular involvement revealed that 52 patients (26%) had the condition in the right eye, 69 patients (34.5%) had the condition in the left eye, and 79 patients (39.5%) exhibited bilateral involvement.

In the group of patients who maintained their scheduled appointments without delays, the median age was 80 years (75–80). At their last pre-COVID-19 appointment, 49 patients (49%) had previously undergone cataract surgery. The median interval between this appointment and their prior visit was 8 (4–8) weeks.

Regarding retinal findings, fluid was detected in 81 patients, and 94 patients required an intravitreal injection at their last pre-lockdown visit. Among them, Aflibercept was administered to 78 patients (83%), Ranibizumab to 8 patients (8.5%), and Bevacizumab to 8 patients (8.5%). The scheduled interval between this appointment and the subsequent one was 8 (4–8) weeks.

At the first appointment following the lockdown, 16 patients (16%) were recommended for cataract surgery. Retinal fluid was detected in 62 patients (62%), and 85 patients (85%) required an IVI. Administered anti-VEGF agents were as follows: Aflibercept in 73 patients (85.9%), Ranibizumab in 8 patients (9.4%), and Bevacizumab in 4 patients (4.7%). The median interval from this post-lockdown appointment to the next follow-up visit was 8 (6–10) weeks.

A comparison of ocular status between the pre-COVID-19 and post-COVID-19 appointments revealed no statistically significant difference in best-corrected visual acuity (BCVA) (0.300 (0.200–0.600) vs. 0.300 (0.200–0.600), *p* = 0.103). However, significant differences were observed in macular thickness across several sectors, as follows: (i) central macular sector (C0), (ii) superior macular sectors (inner ring, S1 and outer ring, S2), (iii) nasal macular sector (inner ring, N1), and (iv) inferior macular sectors (inner ring, I1 and outer ring, I2).

**Figure 2:**
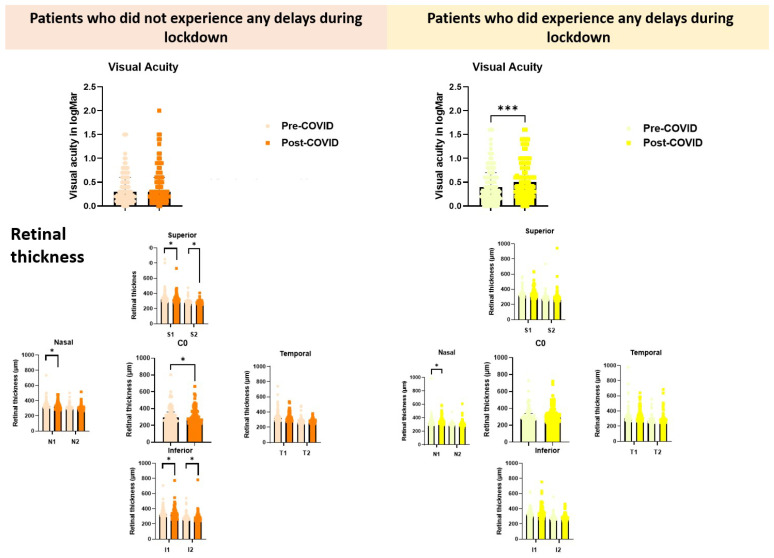
Comparative analysis of visual acuity (logMAR) and retinal thickness across macular regions in patients with and without appointment delays during the COVID-19 lockdown. Orange tones represent patients who did not experience delays, while yellow tones correspond to those who did. Within each colour group, pastel shades indicate measurements taken before the lockdown (Pre-COVID-19), and more saturated tones represent measurements taken after the lockdown (Post-COVID-19). Retinal thickness by OCT is shown for superior (S1, S2, C0), nasal (N1, N2), temporal (T1, T2), and inferior (I1, I2) sectors. * Indicates *p* < 0.05, and *** *p* < 0.001.

These findings are summarised in Table 1 and Figure 2.

In the group of participants who experienced a delay in their scheduled appointments, the median duration of delay was 64.00 days (30.25–104.75), and the median age was 80 years (74–87). At their last pre-lockdown appointment, 44 patients (44%) had previously undergone cataract surgery. The median interval between their previous appointment and the last appointment before the COVID-19 lockdown was 8 weeks (6–10).

Regarding retinal findings, fluid was detected in 65 patients, and 79 patients required an intravitreal injection at their last pre-lockdown visit. Among them, Aflibercept was administered to 63 patients (79.7%), Ranibizumab to 11 patients (13.9%), and Bevacizumab to 5 patients (6.3%). The median time between this appointment and the next scheduled visit was 8 weeks (6–10).

At the first appointment following the lockdown, 6 patients (6%) were recommended for cataract surgery. Retinal fluid was observed in 73 patients (73%), and 92 patients (92%) required an intravitreal injection. The distribution of administered anti-VEGF agents was as follows: Aflibercept in 73 patients (79.3%), Ranibizumab in 12 patients (13%), and Bevacizumab in 7 patients (7.6%). The median interval from this post-lockdown appointment to the next follow-up was 8 weeks (4–10).

Among patients who experienced delays between their last pre-COVID-19 and post-COVID-19 appointments, a statistically significant deterioration in visual acuity was found (0.400 (0.200–0.700) vs. 0.5000 (0.3000–0.9000) (*p* = 0.0002), and a statistically significant increase in macular thickness was observed only in the nasal sector of the inner ring (N1), with values increasing from 324.00 (304.00–358.00) pre-COVID-19 to 337.50 (305.50–375.50) post-COVID-19 (*p* = 0.030). These findings are detailed in Table 1 and Figure 2. Additionally, we compared the age and visual status at the post-COVID-19 appointment between participants who strictly adhered to their scheduled appointments (no delays) and those who experienced delays. While no statistically significant differences were observed in patient age between the two groups, patients who maintained their appointments exhibited significantly higher best-corrected visual acuity (BCVA) compared to those with appointment delays, 0.300 (0.200–0.600) vs. 0.500 (0.300–0.900), (*p* = 0.004).

**Figure 3:**
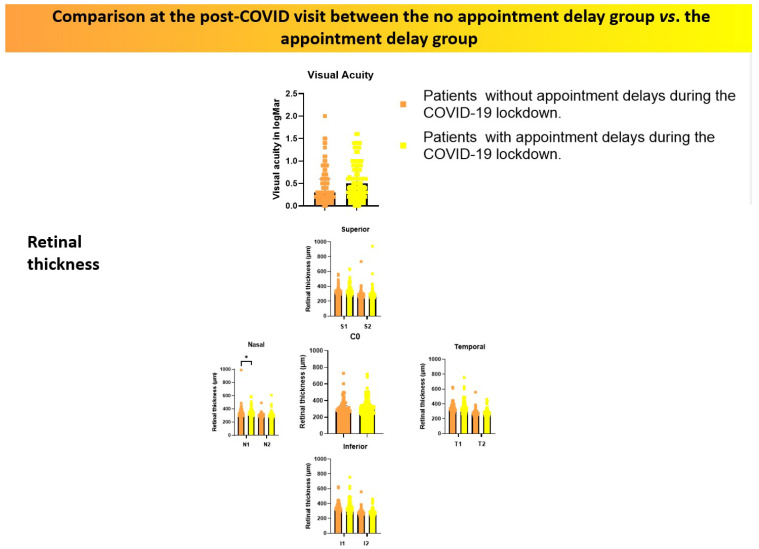
Comparison of visual acuity (logMAR) and retinal thickness measured by OCT between patients who experienced appointment delays and those who did not, assessed at the first post-lockdown clinical visit. Orange tones represent patients without delays, while yellow tones correspond to those with delays. * Indicates *p* < 0.05.

Furthermore, a statistically significant difference was found in macular thickness in the nasal sector of the inner ring (N1) between the two groups, with values of 321.50 (294.00–353.00) in the no-delay group versus 337.50 (305.50–375.50) in the delayed group (*p* = 0.027). These findings are summarised in Table 2 and Figure 3.

**Figure 4:**
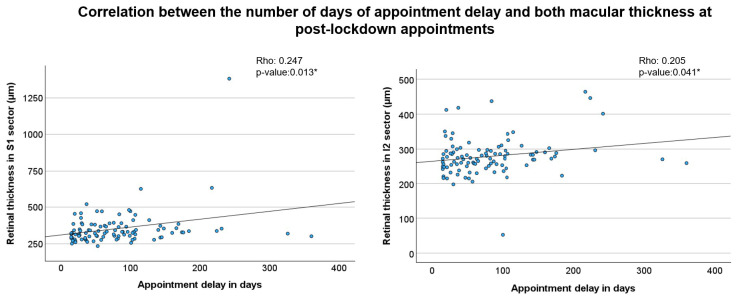
Correlation between appointment delay and macular thickness in post-lockdown visits. The scatter plots show the relationship between the number of days of appointment delay and retinal thickness in two sectors. The left plot (S1 sector) shows a Spearman’s rho of 0.247 (*p* = 0.013 *), and the right plot (I2 sector) shows a Spearman’s rho of 0.205 (*p* = 0.041 *).* Indicates *p* < 0.05.

Finally, we analysed the correlation between the number of days of appointment delay and both BCVA and macular thickness at post-lockdown appointments. While no significant correlation was found between appointment delay and BCVA, a positive and statistically significant correlation was observed between the number of days of delay and macular thickness in multiple macular sectors, (i) S1 and I2 sectors (Figure 4).

These findings indicate that a longer delay in scheduled appointments was associated with increased macular thickness in different regions of the retina.

## 4. Discussion

The COVID-19 pandemic has significantly altered the delivery of outpatient care in healthcare centres, particularly in ophthalmology services. Appointment schedules were adjusted to prioritise high-risk patients based on ophthalmologists’ reviews of medical records [10].

At the WEH Medical Retina clinic, there was a substantial decline in patients attending their routine follow-ups and scheduled intravitreal injections (IVI) of anti-VEGF agents [10]. This trend was also observed in retina clinics of other tertiary hospitals worldwide [14,16,17,18,19]. Additionally, a large proportion of patients with age-related macular degeneration (AMD) have comorbidities such as diabetes, hypertension, or hypercholesterolemia, which are well-established risk factors for severe COVID-19 outcomes [20,21]. Since most AMD patients are elderly, many opted to cancel their appointments due to concerns about contracting COVID-19 or facing travel restrictions [22,23].

Neovascular age-related macular degeneration (nAMD) is one of the most prevalent chronic retinal conditions, and the impact of the pandemic on its management has been widely studied. In response, clinical guidelines and algorithms were developed [10,24], along with recommendations for modified treatment protocols and telemedicine consultations to maintain continuity of care for patients with nAMD while minimising in-person visits [25,26,27].

Our study demonstrates that patients attended their appointments without delays successfully maintained their BCVA following the lockdown. Furthermore, these patients exhibited a statistically significant reduction in macular sector thickness, as assessed by OCT at their first post-lockdown visit. A plausible explanation for this finding is that maintaining regular IVI treatments enhanced their therapeutic effectiveness, leading to a reduction in intra- and/or subretinal fluid and thereby stabilising or improving visual acuity [28].

Conversely, in patients who experienced delays in their appointments, we observed a statistically significant increase in macular thickness in the nasal sector of the inner ring at the first post-lockdown visit. Although other macular sectors exhibited a slight increase in thickness, these changes were not statistically significant. Notably, there were no significant differences in BCVA in this group. Non-compliance with the anti-VEGF IVI regimen has previously been linked to a general decline in vision and increased AMD activity as observed on OCT [29]. Other studies have similarly reported that delays in regular follow-up and IVI treatments due to pandemic-related restrictions resulted in worsening BCVA and retinal structural changes in patients with nAMD [17,30,31]

A comparison of post-lockdown ocular status between the two study groups revealed statistically significant differences only in BCVA, which was higher in patients who had no delays in their appointments. Additionally, patients in this group exhibited lower macular thickness in the nasal sector of the inner ring. These findings are consistent with previous research, which reported that patients with better appointment adherence maintained better visual acuity. That same study also found that patients with higher adherence were generally younger than those who missed appointments during the restriction period [32]. However, in our study, no significant age differences were observed between the two groups.

Moreover, our findings indicate that patients who adhered to their appointments had better baseline visual acuity at their pre-COVID-19 visit compared to those who experienced delays. This suggests that patients with better initial vision may have been more motivated to rigorously follow their treatment regimen.

Finally, we analysed the relationship between the number of days of appointment delay and both BCVA and OCT parameters. Our findings indicate a direct correlation between the length of the delay and retinal thickness in nearly all macular sectors, except for the central macular sector (C0). This suggests that the longer an appointment was postponed, the greater the increase in retinal thickness within the macular region. However, no statistically significant correlation was observed between appointment delay and BCVA in LogMAR. Interestingly, a previous study reported a direct relationship between the intervals between follow-up visits and BCVA outcomes in patients with nAMD [30]

This study has certain limitations. Firstly, it assessed the impact of COVID-19 on the evolution of 200 selected patients from the WEH macula clinic, which may not fully reflect the broader effects of the pandemic on AMD patients in other ophthalmic centres. Additionally, only cases with BCVA measurements and OCT macular analyses available for both the pre-lockdown visit and the first post-lockdown visit were included. In the macula unit, functional and structural assessments, in addition to clinical evaluation, are essential for accurately monitoring disease progression.

Furthermore, it is important to highlight the protocol implemented in the WEH macula unit during the lockdown period. After confinement, patients were recalled based on severity, ensuring that those with the most urgent conditions were prioritised for in-person consultations. This approach may explain why, in our study, the impact of delayed appointments on disease progression was less severe compared to other studies, where patients exhibited a more pronounced decline in BCVA and an increase in retinal disease activity, as evidenced by OCT-measured retinal thickness. This study has an exploratory design, which guided our choice of statistical methods and overall analytical approach. Specifically, we used Spearman’s rank correlation coefficient to assess associations between variables, given the non-parametric nature of the data and the aim to identify general trends rather than causal relationships. In line with this exploratory framework, we adopted a more flexible approach regarding multiple comparisons, prioritising the identification of potential patterns over strict inferential conclusions. While this methodology is appropriate for the study’s objectives, it may limit the depth of statistical inference. We consider these findings to be a preliminary step that may guide the formulation of new hypotheses and inform future studies aimed at validating our results.

## 5. Conclusions

Although our study was conducted on a relatively small sample of patients from a tertiary hospital in London, our findings demonstrate that appointment delays in patients with AMD were associated with a decline in visual acuity compared to those who maintained their scheduled visits.

Despite this, the protocol implemented at WEH suggests that patients can maintain relative disease stability, even in the presence of pathology, provided they receive structured follow-up. However, continuous monitoring and assessment remain essential. Furthermore, it is crucial for clinicians to emphasise to patients the importance of strict adherence to treatment regimens, particularly in preparation for potential future pandemic waves or other disruptions to healthcare services.

From a clinical standpoint, these findings underscore the importance of minimising treatment interruptions in patients with neovascular AMD, as even short-term delays may lead to functional deterioration. This study highlights the need for resilient care pathways that ensure continuity of anti-VEGF therapy, especially in high-risk populations, to prevent irreversible visual loss.

## Figures and Tables

**Table 1:** Analysis of the group of patients who did not experience any delays during lockdown and patients who did experience delays during lockdown.

Patients Who Did Not Experience Any Delays During Lockdown									Patients Who Did Experience Delays During Lockdown							
		Pre-COVID-19 Appointment		Post-COVID-19 Appointment						Pre-COVID-19 Appointment		Post-COVID-19 Appointment				
	*n*	%	Median(IQR)	*n*	%	Median (IQR)	*p*-Value	95%CI	*n*	%	Median (IQR)	*n*	%	Median (IQR)	*p*-Value	95%CI
Age (Years)			80 (75–80)								80 (74–87)					
														64 (30.25–104.75)		
Cataract surgery (No/Yes)	51/49	51/49		84/16	84/16				56/44	56/44		84/16	84/16			
Time between pre-COVID-19 appointments (weeks)			8 (4–8)								8(4–8)					
Macular fluid (No/Yes)	19/81	19/81		38/62	38/62				35/65	35/65		38/62	38/62			
Injection (No/Yes)	6/94	6/94		15/85	15/85				21/79	21/79		15/85	15/85			
Type injection																
Aflibercept	78.00	78.00		73.00	73.00				63	63		73	73			
Ranibizumab	8.00	8.00		8.00	8.00				11	11		8	8			
Bevacizumab	8	8.00		4.00	4.00				5	5		4	4			
Time until next examination (weeks)			8 (4–8)			8 (6–10)	0.013 *	(0.00 to 2.00)			8 (6–10)			8 (4–10)	0.275	(−2.000 to 0.000)
Visual acuity in logMar			0.300 (0.200–0.600)			0.3000 (0.2000–0.6000)	0.103	(−0.008 to 0.093)			0.400 (0.200–0.700)			0.5000 (0.3000–0.9000)	0.0002 ***	(0.000 to 0.1000)
Retinal thickness C0			296.00 (253.25–359.50)			277.50 (245.25–359.75)	0.029 *	(−41.00 to −4.000)			285.50 (252.50–338.75)			300.00 (252.25–363.25)	0.232	(−6.000 to 12.00)
Retinal thickness S1			324.00 (301.00–351.75)			312.50 (291.25–347.25)	0.030 *	(−8.000 to 2.000)			324.00 (293.50–345.00)			328.50 (298.75–373.75)	0.084	(−3.000 to 6.000)
Retinal thickness S2			281.00 (264.00–296.00)			279.00 (257.00–292.00)	0.033 *	(−3.000 to 1.000)			280.50 (263.25–300.00)			282.50 (263.50–305.75)	0.556	(−2.000 to 0.000)
Retinal thickness N1			328.00 (305.00–360.75)			321.50 (294.00–353.00)	0.020 *	(−10.00 to 2.000)			324.00 (304.00–358.00)			337.50 (305.50–375.50)	0.030 *	(−2.000 to 7.000)
Retinal thickness N2			301.00 (283.00–314.00)			298.00 (277.00–310.00)	0.263	(−2.000 to 1.000)			297.00 (276.00–311.00)			299.00 (278.00–318.00)	0.241	(−1.000 to 1.000)
Retinal thickness I1			327.50 (305.00–374.25)			325.00 (285.25–367.75)	0.033 *	(−12.00 to 0.000)			327.50 (293.25–364.75)			327.00 (295.00–380.00)	0.142	(−2.000 to 4.000)
Retinal thickness I2			274.00 (255.00–296.75)			273.00 (250.00–293.50)	0.043 *	(−3.000 to 0.000)			270.50 (250.00–287.75)			272.00 (251.50–294.75)	0.335	(−4.000 to 2.000)
Retinal thickness T1			320.50 (293.00–354.50)			314.00 (286.00–358.50)	0.140	(−9.000 to 3.000)			310.00 (282.00–341.00)			310.50 (282.25–365.00)	0.880	(−2.000 to 1.000)
Retinal thickness T2			273.50 (260.00–285.75)			271.50 (256.75–286.00)	0.147	(−2.000 to 1.000)			268.0 (257.3–290.0)			272.5 (256.3–296.0)	0.999	(−2.000 to 1.000)

IQR: interquartile range. C0: central macular sector; N1: nasal sector if the inner macular ring; I1: inferior sector of the inner macular ring; T1: temporal sector of the inner macular ring; S1: superior sector of the inner macular ring; N2: nasal sector of the outer macular ring. I2: inferior sector of the outer macular ring; T2: temporal sector of the outer macular ring; S2: superior sector of the outer macular ring. * Indicates *p* < 0.05; *** *p* < 0.001.

**Table 2:** Comparison at the post-COVID-19 visit between the no appointment delay group vs. the appointment delay group.

	Patients with no Delays in Appointments	Patients with Late Appointments		
	Median (IQR)	Median(IQR)	*p*-Value	95%CI
Age (years)	80 (75–80)	80 (74–87)	0.928	(−2.000 to 2.000)
Visual acuity in logMar	0.3000 (0.2000–0.6000)	0.5000 (0.3000–0.9000)	0.004 **	(0.000 to 0.2000)
Retinal thickness C0	277.50 (245.25–359.75)	300.00 (252.25–363.25)	0.116	(−4.000 to 38.00)
Retinal thickness S1	312.50 (291.25–347.25)	328.50 (298.75–373.75)	0.064	(−1.000 to 26.00)
Retinal thickness S2	279.00 (257.00–292.00)	282.50 (263.50–305.75)	0.091	(−1.000 to 15.00)
Retinal thickness N1	321.50 (294.00–353.00)	337.50 (305.50–375.50)	0.027 *	(2.000 to 28.00)
Retinal thickness N2	298.00 (277.00–310.00)	299.00 (278.00–318.00)	0.457	(−5.000 to 11.00)
Retinal thickness I1	325.00 (285.25–367.75)	327.00 (295.00–380.00)	0.402	(−9.000 to 24.00)
Retinal thickness I2	273.00 (250.00–293.50)	272.00 (251.50–294.75)	0.708	(−8.000 to 12.00)
Retinal thickness T1	314.00 (286.00–358.50)	310.50 (282.25–365.00)	0.828	(−14.00 to 17.00)
Retinal thickness T2	271.50 (256.75–286.00)	272.5 (256.3–296.0)	0.315	(−4.000 to 13.00)

IQR: interquartile range. C0: central macular sector; N1: nasal sector if the inner macular ring; I1: inferior sector of the inner macular ring; T1: temporal sector of the inner macular ring; S1: superior sector of the inner macular ring; N2: nasal sector of the outer macular ring. I2: inferior sector of the outer macular ring; T2: temporal sector of the outer macular ring; S2: superior sector of the outer macular ring. * Indicates *p* < 0.05, and ** *p* < 0.01.

## Data Availability

Data are available upon request from the corresponding author.

## Abbreviations

The following abbreviations are used in this manuscript:

AMD	Age-related macular degeneration
BCVA	Best corrected visual acuity
OCT	Optical coherence tomography
